# Supplementary material for: Analysis of PPARγ Signaling Activity in Psoriasis
Source: Int J Mol Sci. 2021 Aug 10;22(16):8603. doi: 10.3390/ijms22168603 (PMC8395241; doi:10.3390/ijms22168603)
Supplement: Supplementary file 1 [file ijms-22-08603-s001.zip › Supplemental materials_Analysis of PPARg signaling activity in psoriasis/Pathway models/Models images and html files/Interleukin-17 and interleukin-22 signaling in psoriasis/10358.html]

CCL20


# Protein CCL20

|  |  |
| --- | --- |
| URN | urn:agi-llid:6364 |
| Total Entities | 0 |
| Connectivity | 2291 |
| Name | CCL20 |
| Description | chemokine (C-C motif) ligand 20 |
| Notes | This antimicrobial gene belongs to the subfamily of small cytokine CC genes. Cytokines are a family of secreted proteins involved in immunoregulatory and inflammatory processes. The CC cytokines are proteins characterized by two adjacent cysteines. The protein encoded by this gene displays chemotactic activity for lymphocytes and can repress proliferation of myeloid progenitors. Two transcript variants encoding different isoforms have been found for this gene. [provided by RefSeq, Sep 2014] |
| Primary Cell Localization | Extracellular |
| Class | Ligand |

---

|  |  |
| --- | --- |
| Pathway | CC Chemokine Receptor Signaling |
|  | Plasmin Effects in Inflammation |
|  | IL17 Signaling in Psoriasis |
|  | Proteins Involved in Psoriasis |
|  | Proteins Involved in Diabetic Nephropathy |
|  | Th17-Cell Activation in Crohn's Disease |
|  | Proteins Involved in Inflammatory Bowel Diseases |
|  | Proteins Involved in Helicobacter Infections |
|  | Proteins Involved in Ulcerative Colitis |
|  | Hodgkin and Reed-Sternberg Cells in Hodgkin Lymphoma |
|  | Hodgkin Lymphoma |
|  | Osteoclast Activation in Multiple Myeloma |
|  | IL17F Signaling in Bronchial Epithelial Cell in Asthma |
|  | Th17-Cell Differentiation in Asthma |
|  | Keratinocyte Activation in Psoriatic Arthritis |
|  | TLR2 Induced Synovial Fibroblast Activation in Rheumatoid Arthritis |
|  | Proteins Involved in Neuroblastoma |
|  | Subepithelial Fibroblasts in Middle Ear Cholesteatoma |
|  | Cytokines Trigger Otitis Media |
|  | T-Cell Cytotoxic Activation |
|  | Plasmin Role in Glomerulonephritis |
|  | HMGB1 Expression Targets |
|  | EGFR -> Expression Targets in Skin |
|  | DLL4 Expression Targets |
|  | FASLG Expression Targets |
|  | TNF -> NF-kB Expression Targets |
|  | TNFSF10 Expression Targets |
|  | CD40LG -> NF-kB/ELK/SRF -> CREB/NFATC Expression Targets |
|  | IL1B Expression Targets |
|  | IL1A Expression Targets |
|  | pprarg neg, uknown targets, ps-positive |
|  | prarg neg,ukn expres targets, ps-positive |
|  | PPARG negative targets |
|  | pparg expr target\_regulators |
|  | Neighbors of disease exacerbation |
|  | Neighbors of tumor progression |
|  | Neighbors of cancer progression |
|  | Neighbors of inflammatory status |
|  | Neighbors of overall survival |
|  | Neighbors of disease severity |
|  | Neighbors of tumor size |
|  | Neighbors of lesion size |
|  | Neighbors of mortality |
|  | Neighbors of survival time |
|  | Neighbors of functional recovery |
|  | Neighbors of therapeutic efficacy |
|  | Neighbors of tumor regression |
|  | Neighbors of recurrence-free survival |
|  | Neighbors of disease-free survival |
|  | Neighbors of tumor response |
|  | Neighbors of long-term survival |
|  | Neighbors of bone mass |
|  | Neighbors of neutrophil count |
|  | Neighbors of viral load |
|  | Neighbors of vascular density |
|  | Neighbors of neurological outcome |
|  | Neighbors of recurrence rate |
|  | Neighbors of skin thickness |
|  | Neighbors of disease exacerbation |
|  | Neighbors of tumor progression |
|  | Neighbors of cancer progression |
|  | Neighbors of inflammatory status |
|  | Neighbors of overall survival |
|  | Neighbors of disease severity |
|  | Neighbors of tumor size |
|  | Neighbors of lesion size |
|  | Neighbors of mortality |
|  | Neighbors of survival time |
|  | Neighbors of functional recovery |
|  | Neighbors of therapeutic efficacy |
|  | Neighbors of tumor regression |
|  | Neighbors of recurrence-free survival |
|  | Neighbors of disease-free survival |
|  | Neighbors of tumor response |
|  | Neighbors of long-term survival |
|  | Neighbors of bone mass |
|  | Neighbors of neutrophil count |
|  | Neighbors of viral load |
|  | Neighbors of vascular density |
|  | Neighbors of neurological outcome |
|  | Neighbors of recurrence rate |
|  | Neighbors of skin thickness |
|  | Neighbors of cell development |
|  | Neighbors of immune response |
|  | Neighbors of cell population |
|  | Neighbors of T-cell development |
|  | Neighbors of inflammatory response |
|  | Neighbors of wound healing |
|  | Neighbors of angiogenesis |
|  | Neighbors of innate immune response |
|  | Neighbors of cell function |
|  | Neighbors of cell formation |
|  | Neighbors of adaptive immune response |
|  | Neighbors of osteoclast development |
|  | Neighbors of epithelial to mesenchymal transition |
|  | Neighbors of immunity |
|  | Neighbors of chemotaxis |
|  | Neighbors of dendritic cell differentiation |
|  | Neighbors of cell survival |
|  | Neighbors of osteoclast differentiation |
|  | Neighbors of cell phenotype |
|  | Neighbors of cell adhesion |
|  | Neighbors of fibrogenesis |
|  | Neighbors of tumor growth |
|  | Neighbors of dendritic cell development |
|  | Neighbors of cell count |
|  | Neighbors of cell infiltration |
|  | Neighbors of cell damage |
|  | Neighbors of pregnancy |
|  | Neighbors of monocyte differentiation |
|  | Neighbors of cell motility |
|  | Neighbors of bone resorption |
|  | Neighbors of monocyte migration |
|  | Neighbors of macrophage migration |
|  | Neighbors of neutrophil recruitment |
|  | Neighbors of cell migration |
|  | Neighbors of hepatic regeneration |
|  | Neighbors of transendothelial migration |
|  | Neighbors of immune system activation |
|  | Neighbors of leukocyte migration |
|  | Neighbors of leukocyte recruitment |
|  | Neighbors of osteoclast formation |
|  | Neighbors of monocyte recruitment |
|  | Neighbors of cell invasion |
|  | Neighbors of bone remodeling |
|  | Neighbors of T-cell function |
|  | Neighbors of immune system function |
|  | Neighbors of monocyte adhesion |
|  | Neighbors of tumor immunity |
|  | Neighbors of neuroprotection |
|  | Neighbors of cell growth |
|  | Neighbors of ROS generation |
|  | Neighbors of first trimester pregnancy |
|  | Neighbors of cancer cell growth |
|  | Neighbors of sensitization |
|  | Neighbors of hemato-encephalic barrier |
|  | Neighbors of cellular immune response |
|  | Neighbors of cellular senescence |
|  | Neighbors of colony formation |
|  | Neighbors of cell differentiation |
|  | Neighbors of keratinocyte proliferation |
|  | Neighbors of psoriasis |
|  | Neighbors of rheumatoid arthritis |
|  | Neighbors of atherosclerosis |
|  | Neighbors of fibrosis |
|  | Neighbors of colitis |
|  | Neighbors of arthritis |
|  | Neighbors of inflammatory bowel disease |
|  | Neighbors of injury |
|  | Neighbors of atherogenesis |
|  | Neighbors of metastasis |
|  | Neighbors of carcinogenesis |
|  | Neighbors of multiple sclerosis |
|  | Neighbors of chronic inflammation |
|  | Neighbors of inflammation |
|  | Neighbors of tumor microenvironment |
|  | Neighbors of pathological angiogenesis |
|  | Neighbors of obesity |
|  | Neighbors of asthma |
|  | Neighbors of pneumonia |
|  | Neighbors of osteoarthritis |
|  | Neighbors of chronic obstructive pulmonary disease |
|  | Neighbors of leukocyte infiltration |
|  | Neighbors of airway inflammation |
|  | Neighbors of hepatitis |
|  | Neighbors of insulin resistance |
|  | Neighbors of infection |
|  | Neighbors of liver injury |
|  | Neighbors of neuroinflammation |
|  | Neighbors of sepsis |
|  | Neighbors of encephalomyelitis |
|  | Neighbors of autoimmune disease |
|  | Neighbors of ischemic stroke |
|  | Neighbors of dermatitis |
|  | Neighbors of neutrophil infiltration |
|  | Neighbors of diabetic nephropathy |
|  | Neighbors of lung injury |
|  | Neighbors of immunopathology |
|  | Neighbors of hepatocellular carcinoma |
|  | Neighbors of atherosclerotic plaque |
|  | Neighbors of experimental autoimmune encephalomyelitis |
|  | Neighbors of colorectal cancer |
|  | Neighbors of breast cancer |
|  | Neighbors of melanoma |
|  | Neighbors of cartilage degeneration |
|  | Neighbors of cancer |
|  | Neighbors of cerebral ischemia |
|  | Neighbors of neoplasm |
|  | Neighbors of endometriosis |
|  | Neighbors of liver disease |
|  | Neighbors of osteolysis |
|  | Neighbors of ovarian cancer |
|  | Neighbors of pancreatic cancer |
|  | Neighbors of allograft rejection |
|  | Neighbors of epithelial cell |
|  | Neighbors of keratinocyte |
|  | Neighbors of vascular smooth muscle cell |
|  | Neighbors of umbilical vein endothelial cell |
|  | Neighbors of monocyte |
|  | Neighbors of endometrial stromal cell |
|  | Neighbors of alveolar macrophage |
|  | Neighbors of synoviocyte |
|  | Neighbors of macrophage |
|  | Neighbors of adipocyte |
|  | Neighbors of activated monocyte |
|  | Neighbors of fibroblast |
|  | Neighbors of airway epithelial cell |
|  | Neighbors of PBMC |
|  | Neighbors of stromal cell |
|  | Neighbors of airway smooth muscle cell |
|  | Neighbors of osteoblast |
|  | Neighbors of pneumocyte |
|  | Neighbors of microglia |
|  | Neighbors of intestine epithelium cell |
|  | Neighbors of mononuclear cell |
|  | Neighbors of hepatic stellate cell |
|  | Neighbors of hepatocyte |
|  | Neighbors of kidney mesangial cell |
|  | Neighbors of endothelial cell |
|  | Neighbors of dendritic cell |
|  | Neighbors of inflammatory cell |
|  | Neighbors of bone marrow macrophage |
|  | Neighbors of peritoneal macrophage |
|  | Neighbors of neutrophil |
|  | Neighbors of smooth muscle myocyte |
|  | Neighbors of astrocyte |
|  | Neighbors of bone marrow derived dendritic cell |
|  | Neighbors of lung fibroblast |
|  | Neighbors of activated T-cell |
|  | Neighbors of mononuclear phagocyte |
|  | Neighbors of immunocompetent cell |
|  | Neighbors of granulocyte |
|  | Neighbors of cancer-associated fibroblast |
|  | Neighbors of myeloid cell |
|  | Neighbors of stem cell |
|  | Neighbors of tumor infiltrating macrophage |
|  | Neighbors of B-cell |
|  | Neighbors of granulosa cell |
|  | Neighbors of leukocyte |
|  | Neighbors of insulin-secreting cell |
|  | Neighbors of T-cell |
|  | Neighbors of mast cell |
|  | Neighbors of spleen cell |
|  | Neighbors of brain capillary endothelial cell |
|  | Neighbors of neuroglia |
|  | Neighbors of hematopoietic cell |
|  | Neighbors of antigen-presenting cell |
|  | Neighbors of epidermis cell |
|  | Neighbors of lymphocyte |
|  | Neighbors of M2 macrophage |
|  | Neighbors of lung |
|  | Neighbors of liver |
|  | Neighbors of skin |
|  | Neighbors of intestine |
|  | Neighbors of airway |
|  | Neighbors of blood vessel |
|  | Neighbors of gastrointestinal tract |
|  | Neighbors of synovial membrane |
|  | Neighbors of uterus |
|  | Neighbors of periodontium |
|  | Neighbors of brain |
|  | Neighbors of blood vessel wall |
|  | Neighbors of joint |
|  | Neighbors of lymphatic system |
|  | Neighbors of vagina |
|  | Neighbors of periodontal ligament |
|  | Neighbors of ileum |
|  | Neighbors of small intestine |
|  | Neighbors of NF-kB family |
|  | Neighbors of JNK |
|  | Neighbors of IL1 family |
|  | Neighbors of Jun/Fos |
|  | Neighbors of NFKBI |
|  | Neighbors of PKC |
|  | Neighbors of PI3K |
|  | Neighbors of mitogen-activated protein kinase |
|  | Neighbors of TLR |
|  | Neighbors of cytokine |
|  | Neighbors of interleukin |
|  | Neighbors of JAK |
|  | Neighbors of MEK1/2 |
|  | Neighbors of ERK1/2 |
|  | Neighbors of growth factor |
|  | Neighbors of ovalbumin |
|  | Neighbors of LDL |
|  | Neighbors of NF-AT family |
|  | Neighbors of chorionic gonadotropin |
|  | Neighbors of protein tyrosine kinase |
|  | Neighbors of SMAD subfamily |
|  | Neighbors of p38 |
|  | Neighbors of inflammatory cytokine |
|  | Neighbors of IL23 |
|  | Neighbors of histone deacetylase |
|  | Neighbors of IL1R |
|  | Neighbors of IL12 |
|  | Neighbors of Notch |
|  | Neighbors of heat shock protein 90 |
|  | Neighbors of mitogen-activated protein kinase kinase |
|  | Neighbors of non-coding RNA |
|  | Neighbors of PP2A |
|  | Neighbors of PP2B |
|  | Neighbors of ITG |
|  | Neighbors of CD3 complex |
|  | Neighbors of TORC1 |
|  | Neighbors of phosphodiesterase |
|  | Model of PPARG signaling in psoriasis |
|  | PPARG negative regulators and targets |
|  | Model of PPARG related pathways in psoriasis (short version) |
|  | 2\_Interleukin-17 and interleukin-22 signaling in psoriasis |
|  | 4\_1\_Polymorphisms associated with inflammatory bowel diseases |
|  | Interleukin-17 and interleukin-22 signaling in psoriasis |

---

|  |  |
| --- | --- |
| MedScan ID | 6364 |

---

|  |  |
| --- | --- |
| LocusLink ID | 6364 |
|  | 20297 |
|  | 29538 |

---

|  |  |
| --- | --- |
| Alias | C-C motif chemokine 20 |
|  | CC chemokine ST38 |
|  | MIP-3-alpha |
|  | chemokine CCL20 |
|  | CCL20 |
|  | CC chemokine LARC |
|  | OTTMUSP00000017966 |
|  | CCL20 chemokine |
|  | MIP-3as |
|  | chemokine LARC |
|  | SCYA20 |
|  | chemokine (C-C motif) ligand 20 |
|  | CC chemokine MIP 3alpha |
|  | Beta chemokine exodus-1 |
|  | Small inducible cytokine A20 |
|  | LARC chemokine |
|  | OTTMUSP00000017965 |
|  | MIP3A |
|  | ST38 |
|  | exodus I |
|  | small inducible cytokine subfamily A (Cys-Cys), member 20 |
|  | SY20\_HUMAN |
|  | SY20 |
|  | MIP-3[a] |
|  | exodus-1 |
|  | CKb4 |
|  | chemokine MIP 3alpha |
|  | LARC |
|  | C-C motif chemokine ligand 20 |
|  | Liver and activation-regulated chemokine |
|  | macrophage inflammatory protein 3 alpha |
|  | Exodus |
|  | MIP-3a |
|  | small-inducible cytokine A20 |
|  | beta-chemokine exodus-1 |
|  | small inducible cytokine subfamily A20 |

---

|  |  |
| --- | --- |
| GO ID | 0048020 |
|  | 0031731 |
|  | 0008009 |
|  | 0007186 |
|  | 0072678 |
|  | 0035584 |
|  | 0060326 |
|  | 0007267 |
|  | 0071346 |
|  | 0071347 |
|  | 0071356 |
|  | 0070098 |
|  | 0006935 |
|  | 0019221 |
|  | 0042742 |
|  | 0006955 |
|  | 0006954 |
|  | 0048247 |
|  | 0002548 |
|  | 0030593 |
|  | 0070374 |
|  | 0043547 |
|  | 2000406 |
|  | 0007165 |
|  | 0072679 |
|  | 0005576 |
|  | 0005615 |
|  | 0005125 |
|  | 0050726 |
|  | 0051770 |
|  | 0002724 |
|  | 2000319 |
|  | 0071222 |
|  | 0071223 |
|  | 0071310 |
|  | 0010469 |
|  | 0042060 |
|  | 0042466 |

---

|  |  |
| --- | --- |
| KEGG ID | hsa:6364 |
|  | mmu:20297 |
|  | rno:29538 |

---

|  |  |
| --- | --- |
| Organism | Homo sapiens {Organism urn:agi-taxid:9606} |
|  | Mus musculus {Organism urn:agi-taxid:10090} |
|  | Rattus norvegicus {Organism urn:agi-taxid:10116} |
|  | Homo sapiens |
|  | Mus musculus |
|  | Rattus norvegicus |

---

|  |  |
| --- | --- |
| Mouse chromosome position | 1 42.69 cM |
|  | 1 52.0 cM |

---

|  |  |
| --- | --- |
| OMIM ID | 601960 |

---

|  |  |
| --- | --- |
| Rat chromosome position | 9q34 |

---

|  |  |
| --- | --- |
| Hugo ID | 10619 |
|  | HGNC:10619 |

---

|  |  |
| --- | --- |
| Human chromosome position | 2q36.3 |
|  | 2q33-q37 |

---

|  |  |
| --- | --- |
| Swiss-Prot Accession | P78556 |
|  | P78556.1 |
|  | O89093 |
|  | Q642U4 |
|  | Q54AI7 |
|  | O89093.1 |
|  | P97884 |
|  | P97884.1 |
|  | Q53S51 |
|  | Q99664 |
|  | Q9Z1X3 |
|  | F8WHA7 |

---

|  |  |
| --- | --- |
| GenBank ID | NC\_000002 |
|  | NM\_004591 |
|  | NP\_004582 |
|  | NM\_001130046 |
|  | NP\_001123518 |
|  | AC073065 |
|  | AAX93214 |
|  | AY150053 |
|  | AAN28940 |
|  | CH471063 |
|  | EAW70877 |
|  | EAW70878 |
|  | HI574034 |
|  | CBX47506 |
|  | BC020698 |
|  | AAH20698 |
|  | D17012 |
|  | D86955 |
|  | BAA13191 |
|  | U64197 |
|  | AAB61534 |
|  | U77035 |
|  | AAC50943 |
|  | P78556 |
|  | NC\_000067 |
|  | NM\_016960 |
|  | NP\_058656 |
|  | NM\_001159738 |
|  | NP\_001153210 |
|  | AB015137 |
|  | BAA78113 |
|  | AC123676 |
|  | AJ007862 |
|  | CAA07714 |
|  | CH466629 |
|  | EDL02154 |
|  | EDL02155 |
|  | EDL02156 |
|  | AB015136 |
|  | BAA76955 |
|  | AF053313 |
|  | AAC78295 |
|  | AF099052 |
|  | AAC78680 |
|  | AJ222694 |
|  | CAA10948 |
|  | AK079862 |
|  | BC028504 |
|  | AAH28504 |
|  | BX525886 |
|  | BY026108 |
|  | O89093 |
|  | NC\_005108 |
|  | XM\_006245164 |
|  | XP\_006245226 |
|  | NM\_019233 |
|  | NP\_062106 |
|  | AC\_000077 |
|  | AABR07068189 |
|  | AAHX01061198 |
|  | CH474004 |
|  | EDL75527 |
|  | AF053312 |
|  | AAC78294 |
|  | U90447 |
|  | AAB61459 |
|  | P97884 |
|  | NC\_018913 |
|  | AMYH02005926 |
|  | DQ891973 |
|  | ABM82899 |
|  | DQ896841 |
|  | ABM87840 |
|  | XM\_006496447 |
|  | XP\_006496510 |
|  | XM\_006496448 |
|  | XP\_006496511 |
|  | AC\_000023 |
|  | AAHY01007985 |
|  | AC\_000134 |
|  | NT\_005403 |
|  | NW\_921618 |
|  | NW\_001838866 |
|  | AC\_000045 |
|  | NT\_039173 |
|  | NW\_001030684 |
|  | Q54AI7 |
|  | Q642U4 |
|  | NW\_001084884 |
|  | NW\_047816 |
|  | ABBA01050170 |

---

|  |  |
| --- | --- |
| Swiss-Prot ID | CCL20\_HUMAN |
|  | CCL20\_MOUSE |
|  | CCL20\_RAT |
|  | Q642U4\_MOUSE |
|  | Q54AI7\_MOUSE |

---

|  |  |
| --- | --- |
| Cell Localization | Secreted |
|  | Extracellular |

---

|  |  |
| --- | --- |
| Ensembl ID | ENSG00000115009 |
|  | ENSP00000386273.3 |
|  | ENST00000409189.7 |
|  | ENSP00000351671.4 |
|  | ENST00000358813.5 |
|  | ENSMUSG00000026166 |
|  | ENSMUSP00000139923.1 |
|  | ENSMUST00000186832.1 |
|  | ENSMUSP00000109064.2 |
|  | ENSMUST00000113437.8 |
|  | ENSRNOG00000015992 |
|  | ENSRNOP00000021730.1 |
|  | ENSRNOT00000021730.3 |
|  | ENSP00000386273 |
|  | ENST00000409189 |
|  | ENSP00000351671 |
|  | ENST00000358813 |
|  | ENSMUSP00000139923 |
|  | ENSMUST00000186832 |
|  | ENSMUSP00000109064 |
|  | ENSMUST00000113437 |
|  | ENSMUSP00000027351 |
|  | ENSMUST00000027351 |
|  | ENSRNOP00000021730 |
|  | ENSRNOT00000021730 |

---

|  |  |
| --- | --- |
| MGI ID | MGI:1329031 |
|  | 1329031 |

---

|  |  |
| --- | --- |
| RGD ID | 3646 |

---

|  |  |
| --- | --- |
| Unigene ID | Hs.75498 |
|  | Mm.116739 |
|  | Rn.10722 |

---

|  |  |
| --- | --- |
| Homologene ID | 3375 |

---

|  |  |
| --- | --- |
| Shape | Rhomb |

---

|  |  |
| --- | --- |
| IPI ID | IPI00130917 |
|  | IPI00221705 |
|  | IPI00020355 |
|  | IPI00216291 |
|  | IPI00208858 |
|  | IPI00750862 |

---
